# Mixed Erdheim–Chester disease with thoraco-abdominal involvement

**DOI:** 10.1177/20584601251401103

**Published:** 2025-11-20

**Authors:** Thomas Saliba, David Rotzinger, Laura Haefliger, Guillaume Fahrni

**Affiliations:** Radiology Department, Centre Hospitalier Universitaire Vaudois, Lausanne, Switzerland

**Keywords:** langerhans cell histiocytosis, Erdheim–Chester disease, chronic myelomonocytic leukemia, lung cysts, MAPK pathway, mixed histiocytosis

## Abstract

Erdheim–Chester disease (ECD) is a rare non-Langerhans cell histiocytosis. Mixed ECD–Langerhans cell histiocytosis (LCH) is uncommon, with fewer than 200 cases reported. Diagnosis is challenging and relies on clinical, radiological, and histopathological correlation. We present the case of a 61-year-old man with night sweats, weight loss, and recently diagnosed type 2 diabetes. Imaging revealed cystic lung lesions, perirenal infiltration, and circumferential aortic wall thickening. FDG PET-CT demonstrated multifocal hypermetabolism involving lymph nodes, perirenal soft tissues, and the aortic wall, but no bone involvement. These lesions were shown to progress on subsequent imaging. A lymph node and perirenal biopsies confirmed a mixed form of ECD-LCH with BRAFV600 E mutation and associated chronic myelomonocytic leukemia. The patient was started on targeted therapy with cobimetinib, a MEK inhibitor. Mixed ECD-LCH is a rare entity that typically demonstrates more frequent and widespread organ involvement, particularly affecting the lungs. Its clinical and radiological presentation can have features of both disorders, such as bone, lung, kidney, and vascular involvement. The diagnosis is challenging and requires biopsy with histopathology and genetic testing to be confirmed. Treatment is generally targeted therapy guided by the driver mutations that are identified. We present a rare case of mixed ECD-LCH with thoraco-abdominal and pulmonary involvement. Comprehensive diagnostic workup including histopathology and molecular profiling is crucial for accurate diagnosis and initiation of targeted therapy.

## Introduction

Erdheim–Chester disease (ECD) was originally described as ‘Lipid Granulomatosis’ in 1930 by Jakob Erdheim and William Chester.^1^ It is an exceedingly rare non-Langerhans cell histiocytosis with fewer than 500 reported cases as of 2021, though the number has since grown slightly.^2–4^ ECD has been recently classified as a histiocytic neoplasm, due to the recent discovery of BRAF^V600E^ and mitogen-activated protein kinase (MAPK) pathway mutations.^5^ The average ECD sufferer will present at 53 years old, with reports of cases ranging from 7 to 84 years.^1,6^ There is a slight males preponderance (M:F ratio of 3:1), with men typically presenting at a later disease stage than females.^1,6^

Langerhans cell histiocytosis (LCH) is the most common histiocytic disorder resulting from the coalescence of granulomatous lesions and is often linked to MAPK pathway mutations.^7^ LCH is more frequent in children, where the involvement is generally systemic.^7^ In adults it mainly affects the lungs of patients with a history of smoking.^7^ This is in contrast with ECD, where no link between pulmonary involvement and smoking has been established.^8^ Although ECD affects slightly more males, this trend is reversed in mixed ECD–LCH, where more female cases are reported.^1,4,6^

Though ECD and LCH have some clinical and genetic similarities they remain distinct diseases.^9^ Mixed cases exist, with 20% of ECD patients also having LCH.^9^ Furthermore, there can be associations with other myeloproliferative or myelodysplastic diseases.^9^ Nevertheless, mixed forms are rare, with fewer than 200 reported cases as of 2024.^10^

We present a case of a 61-year-old man with a multisystemic mixed ECD–LCH manifesting as pulmonary, aortic, and renal involvement. This case is remarkable for its absence of bone lesions, a finding atypical for this disease.

## Case report

A 61-year-old man was transferred to our hospital with complaints of fatigue and constitutional B-symptoms including night sweats and unintentional weight loss over the preceding month. The patient reported having recently developed submandibular and inguinal lymphadenopathy but denied having any fever. He also reported a recent diagnosis of type 2 diabetes. The patient had a history of pulmonary emphysema and a smoking history of 45 pack-years.

Upon admission to the hospital, a physical examination confirmed the presence of submandibular, axillary and inguinal lymphadenopathy, alongside bilateral pitting oedema of the legs and a mouth ulcer.

**Fig 1.**
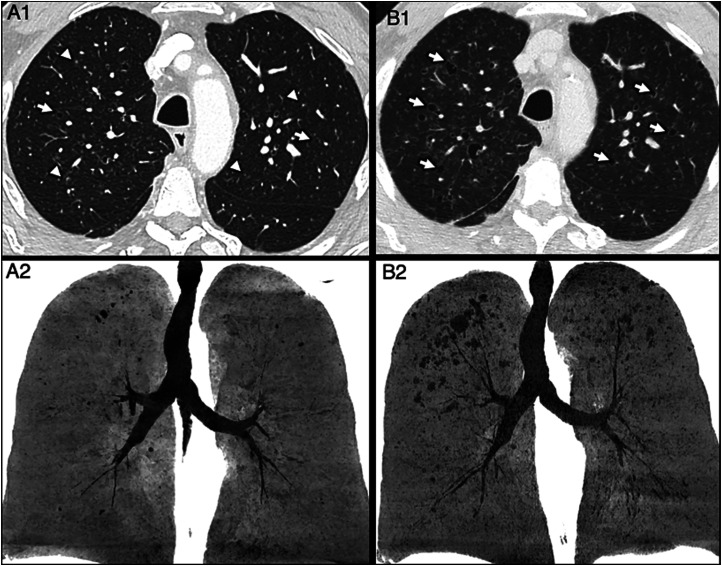
Axial contrast-enhanced CT images of the chest with lung windowing showing small cystic lesions in the initial chest-CT (arrows A1), which can be seen in the upper lung on a minimum intensity projection (MinIP) (A2). On a follow up scan 2 months later, these lesions can be seen to have grown in size and in number (arrows, B1). This is also seen on the MinIP image (B2).

An thoracoabdominal contrast-enhanced CT (CECT) was requested revealing small pulmonary nodules along with small thin-walled cystic lesions in the upper lobes, initially thought to be linked with the patient’s history of lung emphysema (Fig. 1(a)), along with subtle perirenal infiltration. The exam was otherwise unremarkable, and the patient was discharged pending follow-up.

**Fig 2.**
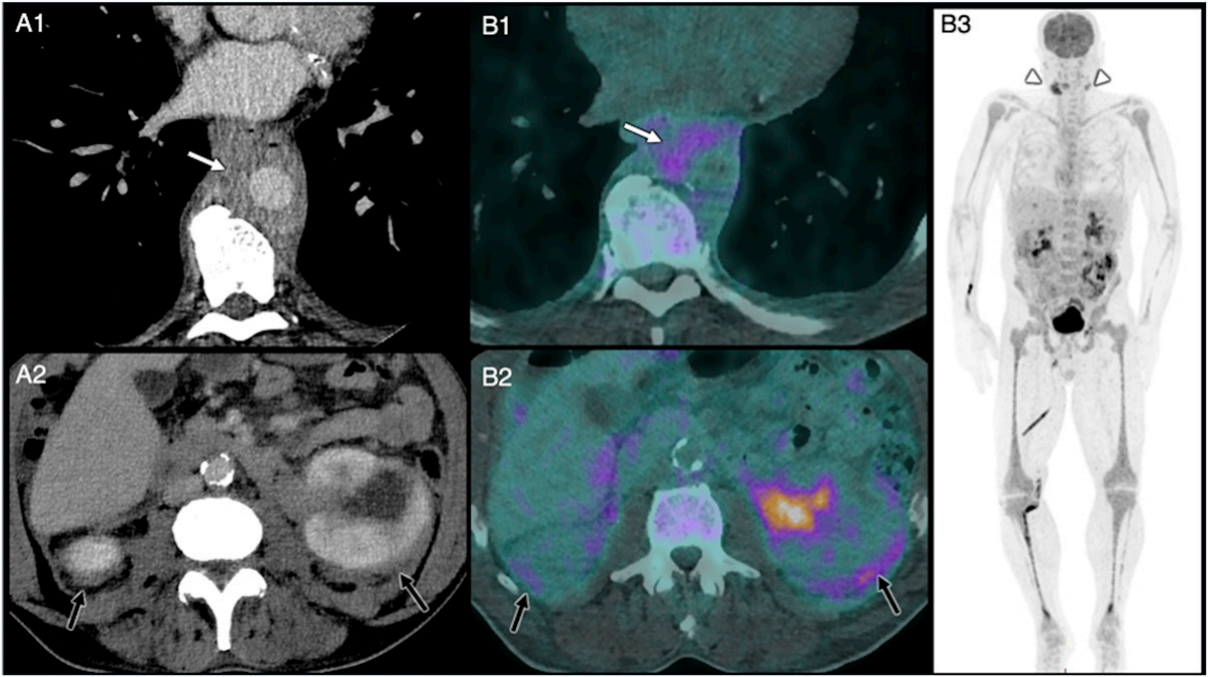
Axial FDG PET-CT with contrast-enhancement images acquired 10 days after the initial CT-scan showing periaortic wall thickening (arrow, A1), which is also hypermetabolic (arrow, B1). Peri-renal and peripyelic infiltration, giving a ‘hairy kidney’ appearance, is also seen (arrow, A2), with hypermetabolism of the area (arrow, B2). A maximum intensity projection of the PET-CT shows numerous hypermetabolic areas that appear dark, with notable neck lymphadenopathy (arrows, B3).

The patient returned for a follow-up CECT exam 2 months later which demonstrated multiple new thin-walled cystic lung lesions with an upper lobe predominance alongside progression of the pre-existing lesions (Fig. 1(b)). There was also progression of the peri-renal and peri-pyelic infiltration as well asaortic wall thickening (Fig. 2(a)). At this point it was concluded that the lung lesions were likely either infectious pneumatoceles or septic emboli and that the patient had concomitant aortitis.

To further the diagnostic workup, a blood test was performed. This revealed that the patient had a comorbid myelodysplastic/myeloproliferative neoplasm, namely myeloproliferative-type chronic myelomonocytic leukemia (MP-CMML) (with a clonal link to LCH disease). The medullogram was hypercellular, with predominance of granulocytes.

FDG PET-CT was requested (Fig. 2(b)) following clinical discovery of suspected lymphadenopathy and leucocytosis, being performed 10 days later. It showed hypermetabolic cervical and inguinal lymphadenopathies (SUVmax 7.8, 10–14 mm short axis), moderate hypermetabolism of tissue infiltration around the aorta (SUVmax around ∼3.8) as well as moderate hypermetabolism of peri-renal infiltration (SUVmax 4).

A biopsy of the neck lymph nodes was thus performed, with histopathological analysis was consistent with LCH and genetic testing demonstrating a BRAF^V600E^ mutation. A biopsy of the peri-renal infiltration was also performed, demonstrating non-Langerhans histiocytic cells infiltration, BRAF^V600E +^, features consistent with ECD^,^ Further biopsies of the medulla of the bone, a mouth ulcer revealed a mixed form of chronic myeloid leukaemia, ECD and LCH.

Upon analysis of the totality of the patient’s symptoms, radiological features and biopsy results, the overall presentation suggested a diagnosis of mixed ECD-LCH.

**Fig 3.**
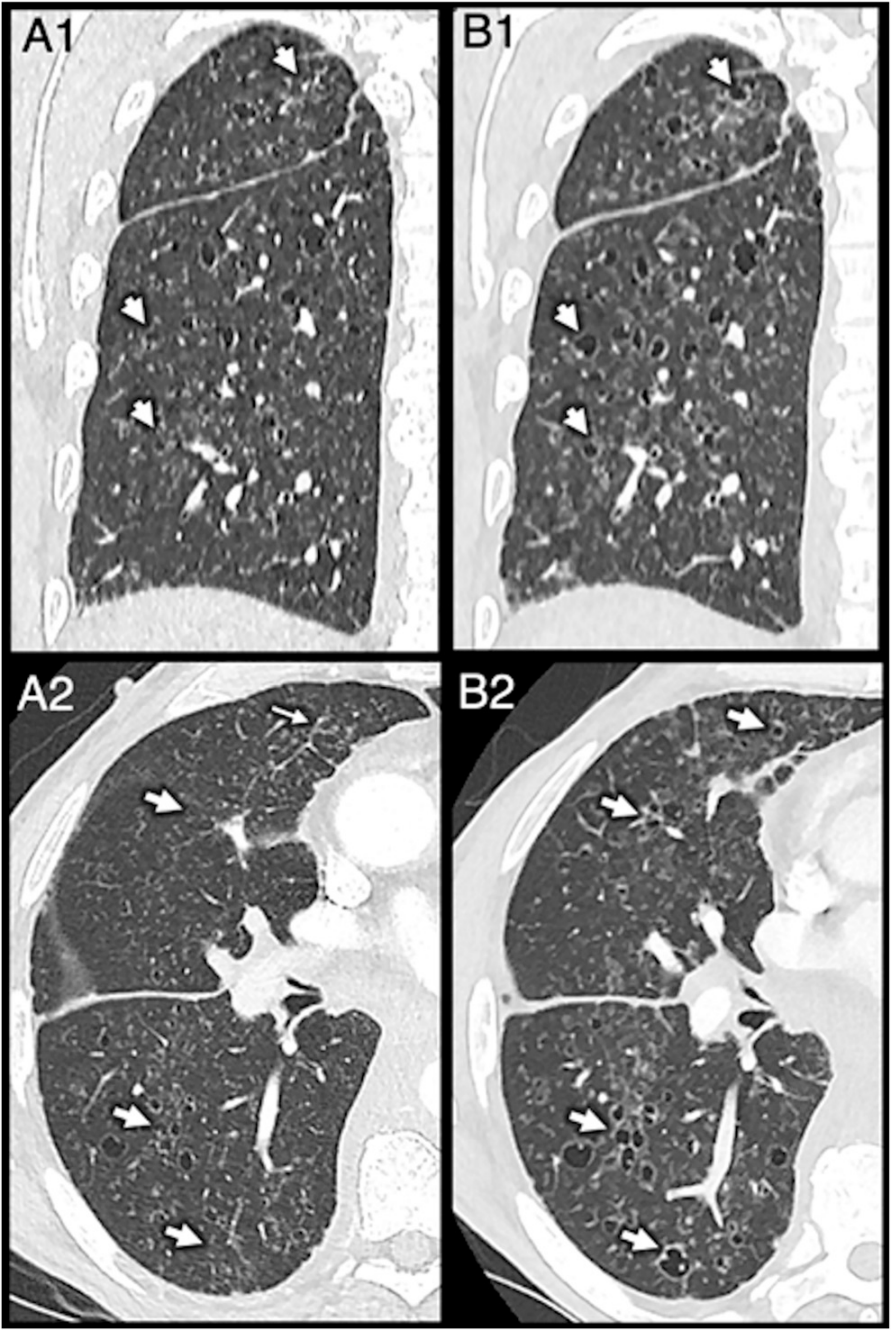
Axial (A1, B1) and coronal (A2, B2) contrast-enhanced images of the chest with lung windowing showing progression in size and number of the cystic lesions from those acquired 2 months after the initial diagnosis (arrows, A1 and A2) compared to imaging acquired 3 months after the initial diagnosis (arrows, B1, B2).

Due to the importance of targeted treatment in these cases, genetic testing was performed to find a suitable target. This revealed a loss of 12p13.31p13.1 (ETV6) (90%). Analysis of fragments also showed ASXL1 c.1934dup, p. Gly646Trpfs*12 (variant allele frequency (VAF) 33%). Finally, DNA sequencing showed variant alleles of CBL (VAF 6.2%), NRAS (VAF 39%), PTPN11 (VAF 6.6%), SETBP1 (VAF 53%), SRSF2 (VAF 53%) and TET2 (VAF 47%). A follow-up thoracic CT scan performed 1 month later showed further progression of the pulmonary cystic lesions (Fig. 3). The patient was started on targeted therapy with a MEK inhibitor (cobimetinib).

**Fig 4.**
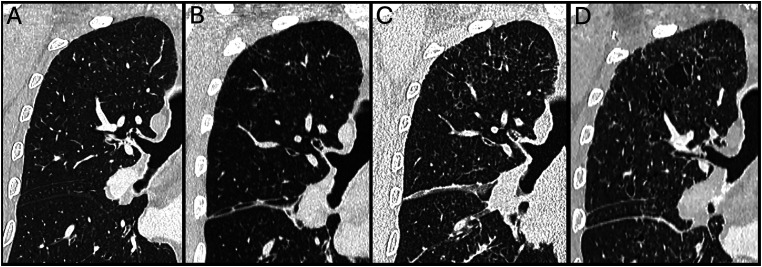
Coronal chest CT images with lung windowing, focused on the upper left lobe, show rapid progression of cystic lesions. (a) Baseline scan; (b) 2 months later; (c) 2 months and 10 days and (d) 3 months demonstrating a progressive increase in the number and size of thin-walled cysts.

A follow-up PET-CT was performed 5 months after the initial diagnosis (2 months after the initiation of treatment). This showed a progression of the disease with an increase of the thin-walled thoracic cysts (Fig. 4). This was accompanied by an increase of the metabolic activity of the perirenal of the peri-renal infiltration (SUVmax 15.7). The appearance and hypermetabolism of the peri-aortic thickening was unchanged.

Due to the rarity of patient’s case, a panel of external experts were consulted regarding the treatment. Following this, it was decided that the patient should be switched to 5-azacytidine treatment after 2 months of MEK-inhibitor bridge treatment. Furthermore, it was recommended that the patient undergo surveillance by fundoscopy and transthoracic echocardiogram to check for left ventricular failure in view of receiving an allograft for the myeloid leukaemia. Furthermore, it was decided to follow-up by PET-scanner every 3 months. Finally, it was decided that MEK inhibitors were to be continued after the allograft had been performed.

The patient continues to undergo treatment and regular follow-ups according to this treatment plan.

## Discussion

Our patient presented with a rare constellation of findings, mixed ECD–LCH with concomitant CMML and progressive cystic lung lesions in the absence of bone involvement. This combination underscores both the diagnostic and therapeutic challenges of histiocytic neoplasms with overlapping myeloid features.

ECD and LCH are both neoplastic diseases, originating from myeloid hematopoietic progenitor cells.^7^ However, despite their common origin, cases of mixed ECD-LCH are rare, with fewer than 200 reported in the literature as of 2024.^4,10^

In cases of ECD, the diagnosis is challenging and relies on the correlation of clinical, radiological, and histopathology findings.^11^ The consensus diagnostic criteria for ECD include xanthogranulomatous lesions with CD68+/CD1a-histiocytes with a fibrosis alongside inflammatory cells and radiographical findings of bilateral and symmetric osteosclerosis in the long bones of lower extremities.^11,12^ Biopsy is recommended to obtain histology and establish the lesion’s genetic profile.^13^ ECD has a variety of histological presentations but is generally characterised by proliferation of mature histiocytes surrounded by inflammatory stroma.^5^ The cellular composition can be heterogeneous and xanthomatous histiocytes will not always be present, making the histological diagnosis challenging.^5^ In cases where the histopathology is unclear, findings of BRAF and MAPK mutations reinforces the diagnosis.^11,14^

Mixed forms of ECD and LCH can present with various features from both diseases. The organs affected by a mixed form will depend on whether ECD or LCH features dominate in a given patient.^15^ When multiple organs are affected, the patient is more likely to have an ECD dominant form, whereas LCH forms will tend to only affect single organs.^15^ Each organ will typically only present with features of either ECD or LCH, though rare mixed forms within a single organ do exist.^12^ It essential that radiologists be aware of potential co-existence of the disease if patterns consistent with both ECD and LCH are found in a single patient.^12^

The coexistence of myeloid neoplasms with histiocytic disorders such as ECD and LCH is increasingly recognized, supporting a shared clonal hematopoietic origin. Though rare, it can be explained by the clonal mutation convergence in genes that activate the MAPK pathway.^16^ Furthermore, there are several mutations which are known to co-exist, including TET2 and KRAS amongst others.^17^ This reinforces the hypothesis that mixed ECD–LCH and CMML may arise from early myeloid progenitors harbouring MAPK pathway mutations, which subsequently drive both histiocytic proliferation and myelomonocytic expansion.^17^ Although reports of CMML coexisting with ECD are exceedingly rare, cases of LCH with concomitant CMML have been described, often without bone involvement, mirroring our patient’s presentation.^17^ Another salient feature is skin involvement, though our patient only had limited mouth ulcers and did not have the extensive skin lesions described in other cases.^17^ Due to the potential link between the two conditions, it has been suggested that patients who are diagnosed with LCH should systematically undergo blood tests to search for concomitant CMML, both upon initial presentation and follow-up.^17^

Lung involvement in ECD occurs in 43–91% of cases.^18,19^ Radiological features of lung involvement will often manifest as interstitial lung disease, ranging from mild to severe, alongside nodules, ground glass opacities, interlobular fissure thickening and pleural involvement.^19^ Another salient feature is the development of and sporadic thin-walled cysts of under 15 mm in diameter.^19^

Lung involvement is also common in LCH, affecting 89% of patients in one cohort.^20^ The disease typically manifests with granulomas that progress to thick-walled cysts, which subsequently evolve into thin-walled cysts, similar in aspect to those seen in ECD.^21^ The natural progression of these cysts is that they will be round and small initially but typically become much larger and irregular as the disease progresses.^21^

When comparing the two diseases lung involvement was nearly twice as frequent in mixed forms (76.8%) compared to simple ECD (43.6%).^4^ Despite this more aggressive and widespread disease phenotype, long-term survival outcomes were comparable between the two groups.

Our patient has a very rare presentation in that they had no bone involvement, a feature that is commonly reported in the literature. In general, bone involvement is nearly universal in ECD, with over 95% of patients being affected, though not all are symptomatic.^11^ These lesions are pathognomonic and include symmetrical cortical osteosclerosis of the diaphysis and the metaphysis of the long bones of the legs and around the knee.^11^

Conversely, our patient had the typical retroperitoneal manifestation of soft-tissue masses in peri-renal and para-renal space (‘hairy peri renal infiltration’), with extension to the renal pelvis.^8^ Around half of the patients with these features have pain and dysuria, though this was not reported by our patient. When imaged, the soft mass infiltration is moderately enhancing on CT and MRI with lower sensitivity on FDG PET-CT. In more chronic disease, it may result in renal atrophy and ureteral proximal obstruction.^1,4,8^

ECD is also known to affect other sites including the heart, skin, central nervous system and eyes amongst others.^14^ Neurological deficits are varied and include endocrinological manifestations, cerebral ataxia and neurodegeneration.^1,4^ Cardiac involvement may manifest as pericarditis with pericardial effusion, ECG anomalies or may even present as a cardiac pseudotumor (myocardial infiltration with predilection for the right atrium) and accounts for significant mortality.^1^

Patients will often have abnormal laboratory results, with 80% having elevated CRP, though elevated cytokines are also frequent.^14^

Treatment is often systemic except in cases of involvement of a single non-vital organ or patients with few symptoms.^13^ Due to the mutations that cause the diseases being identical, mixed ECD-LCH treatments are the same.^4^ Targeted BRAF inhibitor therapy has become the first line of treatment, especially in cases of cardiac or neurological involvement.^13^ In patients without the BRAF mutation, MAPK pathway inhibitors may be considered.^13^ When patients have neither mutation, IFN-α or PEG–IFN-α therapies can be used, though response rates vary depending on the affected organs.^13^ Finally, cytokine-directed therapy, steroids and immunosuppressants are alternative therapy option.^13^ Surgery and radiotherapy are generally not recommended due to the multifocal nature of the disease and the fact that ECD is not radiosensitive.^13^

For ECD, the 1-year survival rate is reported as 96%, dropping to 68% at 5 years.^18^ However, the prognosis of patients with pulmonary ECD is not necessarily poor, with some patients having a stable disease course and survival comparable to those without pulmonary involvement, though this is debated.^18,22^ Nevertheless, a majority of patients will die of progressive lung disease.^23^

Outcomes for mixed ECD-LCH are similar, reported as 65% survival at 6 years according to one study, with most deaths being attributable to nervous or lung involvement.^4^

In conclusion, this case highlights the diagnostic and clinical complexity of mixed ECD and LCH, particularly when occurring in conjunction with a myeloid neoplasm such as chronic myelomonocytic leukaemia. The shared mutational profile supports a common clonal origin and underscores the importance of integrating molecular findings into diagnostic evaluation. Our patient’s unusual absence of bone involvement, coupled with progressive cystic lung disease, illustrates the heterogeneous nature and unpredictable course of mixed histiocytosis. Comprehensive multidisciplinary assessment, including imaging, histopathology, and molecular profiling, is essential to establish the diagnosis and guide targeted therapy. Continued reporting of such rare cases is vital to improving our understanding of disease mechanisms, prognostic factors, and optimal treatment strategies for this complex entity.^24^

## Consent-for-publication

Written informed consent was obtained for anonymised patient information to be used for scientific purposes via the hospital’s standard informed consent document. No additional ethics committee was required according to the hospital’s policy.

## References

[1] Mazor RD Manevich-Mazor M Shoenfeld Y . Erdheim-chester disease: a comprehensive review of the literature. Orphanet J Rare Dis 2013; 8: 137. 24011030 10.1186/1750-1172-8-137 PMC3849848

[2] Benson JC Vaubel R Ebne BA , et al. Erdheim-chester disease. AJNR Am J Neuroradiol 2023; 44: 505–510. 36997288 10.3174/ajnr.A7832 PMC10171379

[3] Popovic A Curtiss C Damron TA . Solitary radiolucent erdheim-chester disease: a case report and literature review. Open Orthop J 2022; 15: 77–82.

[4] Pegoraro F Papo M Cohen-Aubart F , et al. Long-term outcome and prognosis of mixed histiocytosis (Erdheim-Chester disease and langerhans cell histiocytosis). eClinicalMedicine 2024; 73: 102658. 38841707 10.1016/j.eclinm.2024.102658 PMC11152896

[5] Ozkaya N Rosenblum MK Durham BH , et al. The histopathology of Erdheim-Chester disease: a comprehensive review of a molecularly characterized cohort. Mod Pathol 2018; 31: 581–597. 29192649 10.1038/modpathol.2017.160 PMC6718953

[6] Cavalli G Guglielmi B Berti A , et al. The multifaceted clinical presentations and manifestations of Erdheim–Chester disease: comprehensive review of the literature and of 10 new cases. Ann Rheum Dis 2013; 72: 1691–1695. 23396641 10.1136/annrheumdis-2012-202542

[7] Tillotson CV Reynolds SB Patel BC . Langerhans Cell Histiocytosis. [Updated 2024 Apr 18]. In: StatPearls [Internet]. Treasure Island (FL): StatPearls Publishing; 2025 Jan. Available from: https://www.ncbi.nlm.nih.gov/books/NBK430885/28613635

[8] Aswani Y Patel A Zhan X , et al. Imaging in Erdheim-Chester disease. Radiographics 2024; 44: e240011. 39172709 10.1148/rg.240011

[9] Emile J-F Abla O Fraitag S , et al. Revised classification of histiocytoses and neoplasms of the macrophage-dendritic cell lineages. Blood 2016; 127: 2672–2681. 26966089 10.1182/blood-2016-01-690636 PMC5161007

[10] Ding Y Chen S Huang G , et al. Overlap syndrome of Erdheim-Chester disease and langerhans cell histiocytosis: a case report. CytoJournal 2024; 21: 59. 39737131 10.25259/Cytojournal_174_2024 PMC11683406

[11] Elbaz Younes I Ellis A Zhang X . Updates on Erdheim-Chester disease. Human Pathol Rep 2022; 28: 300636.

[12] Haroche J Cohen-Aubart F Amoura Z . Erdheim-Chester disease. Blood. 2020 Apr 16;135(16):1311–1318. doi: 10.1182/blood.2019002766. 32107533

[13] Aricò M Girschikofsky M Généreau T , et al. Langerhans cell histiocytosis in adults report from the international registry of the histiocyte society. Eur J Cancer 2003; 39: 2341–2348. 14556926 10.1016/s0959-8049(03)00672-5

[14] Hashimoto K Miyoshi K Mizutani H , et al. Successful lung transplantation for pulmonary disease associated with Erdheim–Chester disease. Ann Thorac Surg 2017; 104: e13–e15. 28633251 10.1016/j.athoracsur.2017.02.020

[15] Wang J Wang F Sun J , et al. Pulmonary manifestations of Erdheim–Chester disease: clinical characteristics, outcomes and comparison with langerhans cell histiocytosis. Br J Haematol 2021; 194: 1024–1033. 34423426 10.1111/bjh.17712

[16] Goyal G Heaney ML Collin M , et al. Erdheim-Chester disease: consensus recommendations for evaluation, diagnosis, and treatment in the molecular era. Blood 2020; 135: 1929–1945. 32187362 10.1182/blood.2019003507

[17] Tsai JW Tsou JH Hung LY , et al. Combined Erdheim-Chester disease and langerhans cell histiocytosis of skin are both monoclonal: a rare case with human androgen-receptor gene analysis. J Am Acad Dermatol 2010; 63: 284–291. 20633799 10.1016/j.jaad.2009.08.013

[18] Pires Y Jokerst CE Panse PM , et al. Combined Erdheim-Chester disease and langerhans cell histiocytosis in the lung: a report of 2 patients with overlap syndrome. AJSP Rev Rep 2020; 25: 33–39.

[19] Braendstrup P Hansen DL Kristensen L et al. Journal of clinical haematology case report concomitant langerhans cell histiocytosis and chronic myelomonocytic leukaemia responding to 5-azacitidine. J Clin Haematol. 2022; 3(2): 61–65.

[20] Chauvel E Emile J-F Garnier A , et al. Chronic myelomonocytic leukemia associated adult Langerhans cell histiocytosis: a descriptive and comparative study. Blood 2024; 144: 6733.

[21] Miao HL Zhao AL Duan MH , et al. Clinical presentation and prognostic analysis of adult patients with langerhans cell histiocytosis with pulmonary involvement. BMC Cancer 2020; 20: 911. 32967635 10.1186/s12885-020-07421-z PMC7513534

[22] Castoldi MC Verrioli A De Juli E , et al. Pulmonary langerhans cell histiocytosis: the many faces of presentation at initial CT scan. Insights Imag 2014; 5: 483–492. 10.1007/s13244-014-0338-0 PMC4141336 24996395

[23] Cohen-Aubart F Emile JF Carrat F , et al. Phenotypes and survival in Erdheim-Chester disease: results from a 165-patient cohort. Am J Hematol 2018; 93: E114–E117. 29396850 10.1002/ajh.25055

[24] Egan AJM Boardman LA Tazelaar HD , et al. Erdheim-Chester disease. Am J Surg Pathol 1999; 23: 17–26. 9888700 10.1097/00000478-199901000-00002

